# Thermal indices for assessing the impact of outdoor thermal environments on human health: a systematic review of epidemiological studies

**DOI:** 10.1007/s00484-025-02948-x

**Published:** 2025-06-02

**Authors:** Katerina Pantavou, Adrien Fillon, Lunzheng Li, Zacharias Maniadis, Georgios K. Nikolopoulos

**Affiliations:** 1 Medical School, University of Cyprus, 1678 Nicosia, Cyprus; 2 Institute for Environmental Research and Sustainable Development, National Observatory of Athens, 15236 Athens, Greece; 3 Department of Economics, University of Cyprus, 1678 Nicosia, Cyprus; 4 Economics Department, University of Southampton, Southampton, SO17 1BJ UK

**Keywords:** Heat stress, Cold stress, Health outcomes, PET, UTCI, Apparent temperature, Heat index, Humidex

## Abstract

The global rise in temperatures contributes to the increase of climate-sensitive diseases. Despite mitigation efforts, temperatures are projected to keep rising, highlighting the need for integrated methods to assess the impact of thermal environments on human health. This study summarizes the existing evidence on the statistical relationships (associations) between thermal indices and health outcomes. Medline, Scopus, and Web of Science were systematically searched until December, 2023 for studies examining the association between thermal indices and health outcomes in outdoor environments (protocol registration: PROSPERO CRD42023412470). The quality of the included studies was assessed using the United States National Institutes of Health Quality Assessment Tool. The search identified 5038 records, with 310 meeting eligibility criteria and examining 1143 associations. These associations represented 51 countries, primarily in North America (n = 448, 39.2%) and Europe (n = 399, 34.9%). Temperate climates (n = 597, 52.2%) were the most frequently examined. Seventeen indices were identified, with Apparent Temperature being the most common (141 publications, 634 associations). Frequently used indices included also Heat Index, Universal Thermal Climate Index, and Physiologically Equivalent Temperature. About half of the associations focused on mortality and half on morbidity. The most frequently examined associations were for diseases of the circulatory system (n = 304, 26.6%), all-cause morbidity/mortality (n = 288, 25.2%), and diseases of the respiratory system (n = 151, 13.2%). Among associations examining heat-related outcomes (n = 882), 57.8% (n = 510) suggested an increased risk of adverse health outcomes as indices increased, while for cold-related outcomes (n = 367), 44.1% (n = 162) suggested an increased risk as indices decreased. This systematic review reveals significant associations between thermal indices and health outcomes, indicating that thermal indices could be valuable tools for public health planning. However, the diversity in methodologies across studies highlights the need for standardization in methodology and reporting, including the reporting of non-significant findings.

**Supplementary Information:**

The online version contains supplementary material available at 10.1007/s00484-025-02948-x.

## Introduction

The climate crisis driven by climate change is accelerating faster than initially expected (IPCC 2023; Hansen et al. 2025). Across the globe, climate-related events are already having severe consequences, leading to widespread damage to both ecosystems and human societies (IPCC 2023; Arnell et al. 2016). Over the past decade, global surface temperatures have risen by approximately 1.1 °C compared to the pre-industrial baseline (1850–1900) (IPCC 2023), with numerous temperature records being broken in the past two years (Daalen et al. 2024; WMO 2024). Climate-sensitive pathogens and disease vectors, such as West Nile virus, Vibrio, dengue, malaria, and leishmaniasis, are thriving in increasingly favorable conditions, resulting in a rise of water-, food-, and pest-borne diseases (Daalen et al. 2024). Heat-related mortality and morbidity have increased (Daalen et al. 2024). Additionally, the risk of non-communicable diseases might be growing due to reduced physical activity, as extreme heat discourages outdoor activities during the hottest hours of the day (Daalen et al. 2024).

Despite a growing body of policies and mitigation strategies since the release of the Fifth Assessment Report of the Intergovernmental Panel on Climate Change (IPCC) in 2014, continued emissions of greenhouse gases are anticipated to push temperatures even higher (IPCC 2023). Models project an increase of at least 1.5 °C within this century, making it difficult to prevent global warming from surpassing the 2 °C threshold (IPCC 2023). As a result, climate-related health impacts are expected to worsen, potentially affecting billions of people worldwide. This growing threat highlights the urgent need for integrated approaches to assess the relationship between thermal environments and human health.

Thermal indices quantify the thermal effect of meteorological conditions on human health and well-being (Freitas and Grigorieva 2015). Thermal indices integrate multiple variables of the ambient environment such as air temperature, humidity, wind speed, and solar radiation. Some of them also incorporate physiological variables, such as age, sex, height, weight and activity to estimate thermal stress or comfort experienced by the human body in a given outdoor environment (Freitas and Grigorieva 2015). They usually provide an output of a thermal dimension (°C), which reflects a level into a scale of human thermal comfort, sensation, or stress (Freitas and Grigorieva 2015). Thermal indices are used because they offer a more comprehensive measure of thermal stress by considering environmental factors beyond air temperature and, in some cases, physiological responses. They are widely applied in meteorology (Napoli et al. 2021), public health (Romaszko et al. 2022a), occupational safety (Flouris et al. 2018), urban planning (Tseliou et al. 2022), and tourism (Zare et al. 2018) to assess and mitigate the effects of extreme thermal conditions. Thermal indices are employed by international meteorological agencies, including the National Oceanic and Atmospheric Administration in United States (NOAA-National Weather Service 2024), the Bureau of Meteorology in Australia (Bureau of Meteorology Australian Goverment (BOM) 2010), and the Hong Kong Observatory (Hong Kong Observatory 2022) in People's Republic of China among others. They serve as valuable tools for assessing how weather conditions affect human thermal comfort, predicting heat- or cold-related health risks, and guiding public health interventions (Napoli et al. 2021; Potchter et al. 2022, 2018).

The literature includes a wide variety of thermal indices, developed using diverse theoretical approaches, integrating different sets of variables, applied in different climates (i.e., warm or cold climates) or thermal conditions (i.e., extremes, non-extremes), and producing diverse outputs such as thermal sensation, comfort levels, or stress. Simple thermal indices—including Apparent Temperature (AT), Heat Index (HI), Humidex (HU), Wet-Bulb Globe Temperature (WBGT), and Wind Chill Temperature (WCT)— are estimated by simple equations using only basic meteorological variables (i.e., air temperature, humidity and wind speed). These indices are commonly used by international meteorological agencies (NOAA-National Weather Service 2024; Bureau of Meteorology Australian Goverment (BOM) 2010; Goverment of Canada 2024) due to their reliance on easily accessible data and calculation, which avoid the need for radiation flux estimates. However, thermo-physiological indices, such as the Physiologically Equivalent Temperature (PET) and the Universal Thermal Climate Index (UTCI), are considered more appropriate for the comprehensive assessment of thermal environments as they account for radiation fluxes and are based on heat balance models of the human body. PET and UTCI already have a wide range of applications including their use in the weather forecasting procedure (Napoli et al. 2021; Potchter et al. 2022).

The aim of this paper is to summarize the existing evidence on the statistical relationship, (i.e., association) between thermal indices and human health. This will help identify the most commonly used indices, thereby contributing to the standardization of information and facilitating easier comparison of results. Moreover, this review aims to popularize the use of thermal indices among medical scientists, public health professionals, epidemiologists, and policymakers. It will also examine which diseases have been studied in relation to thermal indices, aiming to consolidate this information and highlight gaps in the application of thermal indices in medical and public health research.

## Materials and methods

### Search strategy and selection criteria

We conducted a systematic review to examine the association between the thermal indices and health outcomes. We searched Medline (via PubMed), Scopus, and Science Citation Index Expanded, Social Sciences Citation Index and Emerging Sources Citation Index (via Web of Science) from inception to December 31 st, 2023. The search algorithm included the words “heat”, “warm”, “cold”, “cool”, “thermal environment” and “thermal condition” which encompass concepts such as heat waves, cold spells, and extreme warm/cool/thermal conditions. Additionally, the search included the terms “weather”, “climate”, “thermal index” and “biometeorological index”, “equivalent temperature”, “universal thermal climate index”, “predicted mean vote”, “wind chill”, “heat index”, “apparent temperature”, “humidex”, “effective temperature”, “perceived temperature”, “mortality”, “death”, “morbidity”, “hospital, emergency”, “health”, “exposure”, “exhaustion”, “illness”, “disease”. Boolean operators (AND, OR) were used to combine these terms appropriately. The search terms were chosen based on their relevance to the core concepts of thermal environments and health, as well as their ability to capture a broader range of related topics. We also included specific biometeorological indices (e.g., predicted mean vote, perceived temperature, wind chill) guided by their widespread use in existing literature, and health outcomes (e.g., exhaustion) to ensure that relevant studies were not overlooked. The full search strategy can be found in the supplement (Online Resource 1– Search strategy).

The inclusion criteria required studies published in English, concentrating on human subjects. There were not any restrictions related to the publication status of the studies; however, publications such as books, letters, and commentary were not assessed due to their potential inability to provide adequate data for inclusion in our study. Publications focusing on indoor thermal environments were excluded as well as those focusing on injuries. Additionally, studies focusing on physiological responses were omitted, as they did not address medical conditions.

Title, abstract, and full text screening was performed in duplicate by two authors (KP, LL) in Rayyan software (Ouzzani et al. 2016) after having removed the duplicates in the Mendeley reference management software (version 1.19.8, 2008–2020 Mendeley Ltd). Disagreements among reviewers during the screening process were resolved through discussion. If consensus could not be reached, a third team member (AF) was consulted to make the final decision. Consensus was defined as an agreement among reviewers regarding the inclusion or exclusion of a study. The reference lists of the identified articles were screened for additional eligible publications. For articles where the full text could not be retrieved, we contacted their authors, primarily through ResearchGate, to obtain the necessary information.

This systematic review follows a standardized methodology based on a predefined protocol (PROSPERO CRD42023412470). The findings are presented in accordance to the PRISMA (Preferred Reporting Items for Systematic Reviews and Meta-Analyses) guidelines (Online Resource 1– PRISMA Checklist) (Page et al. 2020).

### Data extraction and analysis

Data extraction was performed by 3 authors (KP, AF, LL) using a predefined extraction form in Excel. The extracted information from each eligible publication included first author’s last name, year and journal of publication, examined thermal indices and health outcomes, characteristics of the study populations (e.g., country, city, age), type of meteorological data (e.g., data from stations, gridded data, in-situ measurements) and health data sources (e.g., hospital admissions, emergency visits, ambulance calls, statistical services), type of indices’ measures used in the analysis (e.g., daily/weekly/monthly average, median, maximum, or minimum values), analysis method (e.g., correlation, linear, logistic, negative binomial regression), effect estimate (descriptive assessment, correlation coefficient, odds ratio, relative risk), and whether potential confounders were examined in the analysis (adjusted, not adjusted). The climate of the studied areas was obtained using Koppen classification (Kottek et al. 2006). The health outcomes were classified according to the International Statistical Classification of Diseases and Related Health Problems 11 th Revision (ICD-11). Thermal conditions examined in the included publications were classified as warm and cool based on the seasons they focused on.

The analysis was conducted using a standard software package (Stata, version. 18; StataCorp). A two-sample test of proportions (prtest) was used to compare proportions across different categories, such as mortality versus morbidity, to assess whether the observed differences in the effects of thermal indices on health outcomes were statistically significant.

Quality was assessed using the United States National Institutes of Health (NIH) Quality Assessment Tool for Observational Cohort and Cross-Sectional Studies (NIH 2021). The NIH tool critically appraises the quality of studies using 14 items and focusing on key methodological issues for studies’ internal validity. The NIH tool provides a structured and transparent approach for evaluating study quality, minimizing bias, and improving the reliability of synthesized evidence.

## Results

### Studies and associations

**Fig. 1:**
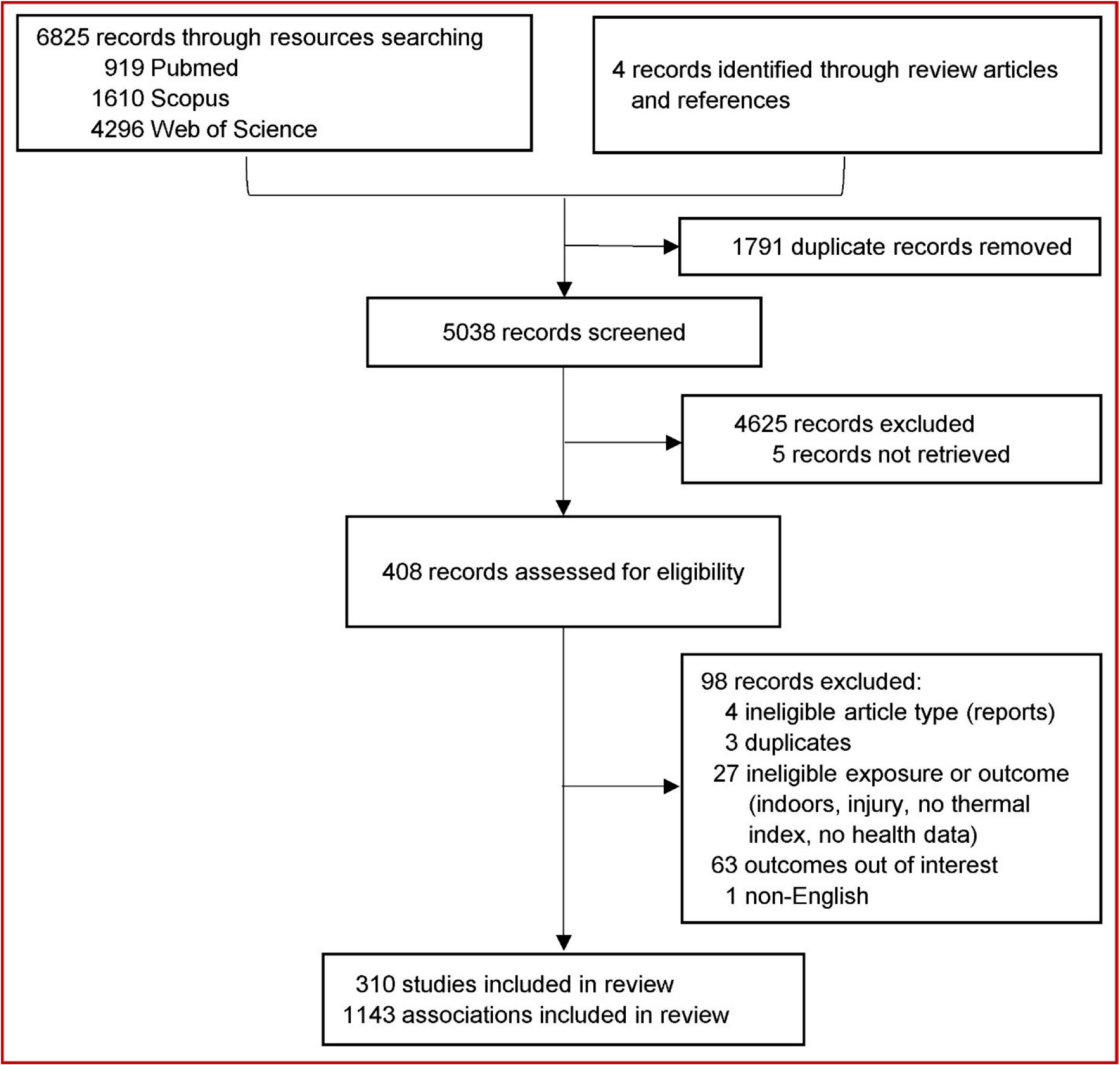
Study selection

The search algorithm across the PubMed, Scopus, and Web of Science databases, identified 6825 records (Fig. 1). Four additional records were found through review articles and reference lists. After removing duplicates, 5038 unique records remained and were screened based on their title and abstract. Of these, 408 met the inclusion criteria and underwent full-text screening. Ultimately, after full-text screening, 310 (O’Neill et al. 2005, 2003; de Donato et al. 2008; Morabito et al. 2012, 2014, 2005; Lee et al. 2016; Choi et al. 2017; Smoyer et al. 2000; Smoyer 1980; Hattis et al. 2012; Xu et al. 2013, 2023; Ostro et al. 2009, 2010; Alessandrini et al. 2011; Basu and Malig 2011; Ragettli et al. 2017; Royé et al. 2020; Davis et al. 2020; Henderson et al. 2013; Chung et al. 2009; Lin et al. 2012, 2013, 2016a, 2009, 2016b; Wichmann 2017; Stanojevic et al. 2014; Analitis et al. 2008; Saha et al. 2014; Bell et al. 2008; Stafoggia et al. 2006, 2009, 2008; Basu et al. 2008, 2015, 2012, 2018a, 2017, 2010, 2018b, 2016; Zanobetti and Schwartz 2008; Baccini et al. 2008; Madrigano et al. 2013; Michelozzi et al. 2006, 2005, 2009; Wiru et al. 2020; Hondula et al. 2012; Schifano et al. 2012, 2009, 2013, 2016; Almeida et al. 2010; D’Ippoliti et al. 2010; Leone et al. 2013; Astrom et al. 2015; Chen et al. 2017; Gronlund et al. 2014, 2020; Ghirardi et al. 2015; Kim et al. 2006, 2018, 2020, 2016; Harlan et al. 2014; Davis and Novicoff 2018; Analitis et al. 2018; Cao et al. 2021; Lee et al. 2021; Yong et al. 2023; Ngarambe et al. 2022; Pantavou et al. 2008, 2011, 2016, 2021; Green et al. 2010; Lim et al. 2022a, 2022b, 2015, 2017, 2021; Meng et al. 2023; Zhan et al. 2023, 2022; Gao et al. 2022; Basu and Ostro 2008; Heidari et al. 2016; Min et al. 2019; Jin et al. 2023; Ohno et al. 1970; Ohno 1970; Roye et al. 2019; Shartova et al. 2018; Zutphen et al. 2012; Wichmann et al. 2011a, 2011b, 2012; Urban and Kyselý 2014; Grjibovski et al. 2012a; Grjibovski et al. 2012b, 2013, 2021; Zhai et al. 2022a, 2023a, 2022b, 2023b; Psistaki et al. 2023; Santurtun et al. 2020; Avalos et al. 2017; Li et al. 2021; Mbanu et al. 2007; Nguyen et al. 2015; Moghadamnia et al. 2018a; Halonen et al. 2011; Zhang et al. 2016, 2023, 2020, 2022, 2017; Moghadamnia et al. 2018b; Shrikhande et al. 2023; Liu et al. 2018a, 2018b, 2021; Telesca et al. 2023; Buehler et al. 2023; Roye et al. 2018; Nick et al. 2022; Zhou et al. 2022, 2023; Krstić and Krstic 2011; Sun et al. 2023; Benmarhnia et al. 2015; Cushing et al. 2022; Veron et al. 2021; Vicedo-Cabrera et al. 2014; Requia et al. 2022; Mohammadi et al. 2019; Porter et al. 1999; Soim et al. 2018, 2017; Hartz et al. 2013, 2012; Ng et al. 2014; Sen and Nag 2019; Milsten et al. 2003; Vassil et al. 2020; Milani et al. 2022; Aguglia et al. 2021; Rammah et al. 2019; Lu et al. 2023; Leung et al. 2008, 2021; Aboubakri et al. 2020; Mohammadi and Karimi 2018; Goncalves et al. 2007; Emelina et al. 2015; Costa et al. 2021; Wenfang et al. 2020; de Sousa Zanotti Stagliorio Coêlho et al. 2010; Gunasekara et al. 2023; Yip et al. 2008; Chien et al. 2016; Perron et al. 2005; Vaidyanathan et al. 2019; Matte et al. 2016; Spangler et al. 2023a; Zottarelli et al. 2021; Rathi and Sodani 2021; Kivimäki et al. 2050; Khatana et al. 2022a, 2022b; Skarha et al. 2022; Monteiro et al. 2013; Weinberger et al. 2021; Burkart et al. 2011, 2013, 2016, 2014; Wellenius et al. 2017; Rosenthal et al. 2014; Sung et al. 2013; Metzger et al. 2010; Levy et al. 2015; Desai et al. 2015; Fritze 2020; Rathi et al. 2017, 2021; Saddique et al. 2021; Williams et al. 2020; Boeke et al. 2010; Deng et al. 2022; Gao et al. 2020; Hahn et al. 2023; Brunetti et al. 2014; Kysely and Huth 2004; Huang et al. 2021; Yin and Wang 2018; Hamilton et al. 2021; Ross et al. 2018; Chu et al. 2022; Tam et al. 2008; Jiao et al. 2023; Son et al. 2019; Carlson et al. 2021; Kranc et al. 2021; Bandala et al. 2019; Shire et al. 2020; Saha et al. 2015; Lewandowski et al. 2022; Na et al. 2013; DeMartini et al. 2014; Grundstein et al. 2012; Bethancourt et al. 2021; Bai et al. 2014; Erickson et al. 2019; Moore et al. 2017; Cloud et al. 2023; Savitz and Hu 2021; Theoharatos et al. 2010; Isaksen et al. 2016, 2015; DeVine et al. 2017; Rainham and Smoyer-Tomic 2003; Conti et al. 2007, 2005; Kegel et al. 2021; Calkins et al. 2016; Ho et al. 2017; Arnold et al. 2022; Zhao et al. 2023; Pan et al. 2019; Bassil et al. 2011; Mastrangelo et al. 2007; Infusino et al. 2021; Thach et al. 2015; Schroeder et al. 2023; Thorsson et al. 2021; Laschewski and Jendritzky 2002; Muthers et al. 2010a, 2010b; Zaninovic et al. 2014; Urban et al. 2019; Matzarakis et al. 2011; Nastos and Matzarakis 2012, 2006, 2008; Dastoorpoor et al. 2022a, 2022b; Zaninovic and Matzarakis 2014; Zemtsov et al. 2020; Sharafkhani et al. 2018; Shiue et al. 2015, 2016a, 2016b, 2016c; Ferrari et al. 2015; Vasconcelos et al. 2013; Roshan et al. 2022a; Roshan et al. 2022b; Caglak 2022; Caglak and Matzarakis 2023; Borsi et al. 2021; Silva and Ribeiro 2012; Dastoorpoor et al. 2021; Pantavou et al. 2020; Kienbacher et al. 2021; Koppe et al. 2011; Schlegel et al. 2020; Garin and Bejaran 2003; Błażejczyk et al. 2018; Blazejczyk et al. 2022; Chau et al. 2022; Urban et al. 2021; Ghada et al. 2021a, 2021b; Romaszko et al. 2017, 2019; Lokys et al. 2018; Romaszko et al. 2022b; Jingesi et al. 2023; Ma et al. 2018; Skutecki et al. 2019; Kuchcik 2021; Lindner-Cendrowska and Bröde 2021; Romaszko-Wojtowicz et al. 2020; Kruger and Nedel 2023; Fallah Ghalhari et al. 2016; Nyadanu et al. 2022a; Nyadanu et al. 2023a, 2023b, 2022b; Cymes et al. 2021; Krzyzewska et al. 2017; Khodadadi et al. 2022; Bonell et al. 2022; Sombatsawat et al. 2023; Pradhan et al. 2019; Meshi et al. 2018; Lewandowski and Shaman 2022; Morris et al. 2019; Wallace et al. 2005; Carder et al. 2005; Oh et al. 2021; Gill et al. 1988; Eng and Mercer 2000; Ohlson et al. 1991) records met the inclusion criteria and were included in this systematic review (Table 1). The included articles examined 1143 associations (Online Resource 2–Table S1) between thermal indices and health outcomes. The earliest publication dates back to 1969 followed by nine studies published until 2002, while the number of relevant publications increased significantly thereafter (Online Resource 1–Figure S1). The articles were distributed across 120 journals with 41.9% (n = 130) being published in journals focusing on environmental sciences, climate and meteorology, 26.8% (n = 83) in journals of epidemiology and public health, and 19% (n = 59) in journals focusing on medical sciences (Online Resource 1–Figure S2).

**Table 1:** Thermal indices identified in this systematic review and characteristics of included studies

Index	Country	Climate	Health outcome
ASV	Greece	Temperate	22 Injury, poisoning or certain other consequences of external causes (Pantavou et al. 2016, 2011)
AT	Argentina, Australia, Brazil, Canada, Chile, Czech Republic, Denmark, Finland, France, Germany, Ghana, Greece, Hungary, India, Iran, Ireland, Israel, Italy, Japan, Kazakhstan, Malaysia, Mexico, People’s Republic of China, Portugal, Republic of China (Taiwan), Republic of Korea, Russia, Serbia, Slovenia, South Africa, Spain, Sweden, Switzerland, Thailand, Tunis, Turkey, United States, United Kingdom	Arid, Continental, Temperate, Tropical	All causes (O’Neill et al. 2005, 2003; de Donato et al. 2008; Morabito et al. 2012, 2014; Lee et al. 2016; Choi et al. 2017; Smoyer et al. 2000; Smoyer 1980; Hattis et al. 2012; Xu et al. 2013; Ostro et al. 2009; Alessandrini et al. 2011; Basu and Malig 2011; Ragettli et al. 2017; Royé et al. 2020; Davis et al. 2020; Henderson et al. 2013; Chung et al. 2009; Lin et al. 2012, 2013; Wichmann 2017; Stanojevic et al. 2014; Analitis et al. 2008; Saha et al. 2014; Bell et al. 2008; Stafoggia et al. 2006, 2009, 2008; Basu et al. 2008, 2015; Zanobetti and Schwartz 2008; Baccini et al. 2008; Madrigano et al. 2013; Michelozzi et al. 2006, 2005; Wiru et al. 2020; Hondula et al. 2012; Schifano et al. 2012, 2009; Almeida et al. 2010; D’Ippoliti et al. 2010; Leone et al. 2013; Astrom et al. 2015; Chen et al. 2017; Gronlund et al. 2014; Ghirardi et al. 2015; Kim et al. 2006; Harlan et al. 2014; Davis and Novicoff 2018; Analitis et al. 2018; Cao et al. 2021; Lee et al. 2021; Yong et al. 2023; Ngarambe et al. 2022) 01 Certain infectious or parasitic diseases (Chen et al. 2017; Green et al. 2010; Lim et al. 2022a, 2022b; Meng et al. 2023; Zhan et al. 2023; Lin et al. 2016a; Ostro et al. 2010; Basu et al. 2012; Gao et al. 2022) 05 Endocrine, nutritional or metabolic diseases (Chen et al. 2017; Harlan et al. 2014; Green et al. 2010; Ostro et al. 2010; Basu et al. 2012; Lim et al. 2015; Basu and Ostro 2008; Heidari et al. 2016) 06 Mental, behavioural or neurodevelopmental disorders (Basu et al. 2018a; Min et al. 2019; Jin et al. 2023) 08 Diseases of the nervous system (Analitis et al. 2008; D’Ippoliti et al. 2010; Chen et al. 2017; Green et al. 2010; Ostro et al. 2010; Basu et al. 2012; Basu and Ostro 2008; Michelozzi et al. 2009; Ohno et al. 1970; Ohno 1970; Zhan et al. 2022; Roye et al. 2019; Shartova et al. 2018; Zutphen et al. 2012; Wichmann et al. 2011a, 2011b; Urban and Kyselý 2014; Grjibovski et al. 2012a; Grjibovski et al. 2012b) 11 Diseases of the circulatory system (Alessandrini et al. 2011; Basu and Malig 2011; Royé et al. 2020; Chung et al. 2009; Analitis et al. 2008; Baccini et al. 2008; Madrigano et al. 2013; Stafoggia et al. 2009; Basu et al. 2015, 2012, 2017; Almeida et al. 2010; D’Ippoliti et al. 2010; Chen et al. 2017; Gronlund et al. 2014; Harlan et al. 2014; Green et al. 2010; Ostro et al. 2010; Basu and Ostro 2008; Michelozzi et al. 2009; Zhan et al. 2022; Shartova et al. 2018; Zutphen et al. 2012; Wichmann et al. 2011a, 2011b, 2012; Urban and Kyselý 2014; Grjibovski et al. 2012a; Grjibovski et al. 2012b; Zhai et al. 2022a, 2023a, 2022b, 2023b; Psistaki et al. 2023; Morabito et al. 2005; Santurtun et al. 2020; Li et al. 2021; Mbanu et al. 2007; Nguyen et al. 2015; Lin et al. 2009, 2016b; Moghadamnia et al. 2018a; Halonen et al. 2011; Zhang et al. 2016; Xu et al. 2023; Moghadamnia et al. 2018b; Shrikhande et al. 2023; Liu et al. 2018a; Telesca et al. 2023; Buehler et al. 2023) 12 Diseases of the respiratory system (Alessandrini et al. 2011; Basu and Malig 2011; Chung et al. 2009; Analitis et al. 2008; Baccini et al. 2008; Stafoggia et al. 2009; Basu et al. 2015, 2012; Almeida et al. 2010; D’Ippoliti et al. 2010; Chen et al. 2017; Gronlund et al. 2014; Harlan et al. 2014; Green et al. 2010; Ostro et al. 2010; Basu and Ostro 2008; Michelozzi et al. 2009; Wichmann et al. 2011a, 2011b; Lin et al. 2009; Roye et al. 2018; Zhang et al. 2023, 2020; Liu et al. 2021; Zhou et al. 2022; Grjibovski et al. 2021) 11 and 12 Diseases of the circulatory and Diseases of the respiratory system (Krstić and Krstic 2011) 14 Diseases of the skin (Sun et al. 2023) 16 Diseases of the genitourinary system (Chen et al. 2017; Gronlund et al. 2014; Harlan et al. 2014; Green et al. 2010; Ostro et al. 2010; Basu et al. 2012; Lim et al. 2015) 18 Pregnancy, childbirth or the puerperium (Avalos et al. 2017; Benmarhnia et al. 2015; Basu et al. 2010; Cushing et al. 2022) 19 Certain conditions originating in the perinatal period (Basu et al. 2015, 2018b; Veron et al. 2021; Schifano et al. 2013, 2016; Vicedo-Cabrera et al. 2014; Requia et al. 2022; Gronlund et al. 2020; Mohammadi et al. 2019; Porter et al. 1999) 20 Developmental anomalies (Basu et al. 2015; Zutphen et al. 2012; Soim et al. 2018, 2017) 21 Symptoms, signs or clinical findings, not elsewhere classified (Basu et al. 2015; Zutphen et al. 2012) 22 Injury, poisoning or certain other consequences of external causes (Harlan et al. 2014; Green et al. 2010; Ostro et al. 2010; Basu et al. 2012; Hartz et al. 2013; Ng et al. 2014; Sen and Nag 2019; Milsten et al. 2003; Vassil et al. 2020; Milani et al. 2022) 23 External causes of morbidity or mortality (Basu et al. 2018a; Aguglia et al. 2021; Grjibovski et al. 2013) 24 Factors influencing health status or contact with health services (Rammah et al. 2019; Basu et al. 2016) Other (non-categorized in one code) (Lu et al. 2023; Zhang et al. 2022)
DI	Greece, Republic of China (Taiwan)	Temperate	All causes (Lin et al. 2013) 22 Injury, poisoning or certain other consequences of external causes (Pantavou et al. 2011)
ET	Brazil, Greece, Iran, People’s Republic of China, Republic of China (Taiwan), Russia, Spain, Sri Lanka	Arid, Continental, Temperate, Tropical	All causes (Lin et al. 2013; Leung et al. 2008; Aboubakri et al. 2020) 11 Diseases of the circulatory system (Psistaki et al. 2023; Santurtun et al. 2020; Mohammadi and Karimi 2018; Goncalves et al. 2007; Emelina et al. 2015; Costa et al. 2021) 12 Diseases of the respiratory system (Nick et al. 2022; Wenfang et al. 2020; de Sousa Zanotti Stagliorio Coêlho et al. 2010) 16 Diseases of the genitourinary system (Gunasekara et al. 2023)
HI	Bangladesh, Czech Republic, Finland, Germany, India, Israel, Italy, Pakistan, People’s Republic of China, Portugal, Republic of China (Taiwan), Republic of Korea, Sri Lanka, United States, Vietnam	Arid, Continental, Temperate, Tropical	All causes (Lin et al. 2012; Yip et al. 2008; Chien et al. 2016; Perron et al. 2005; Vaidyanathan et al. 2019; Matte et al. 2016; Spangler et al. 2023a; Liu et al. 2018b; Zottarelli et al. 2021; Rathi and Sodani 2021; Kivimäki et al. 2050; Khatana et al. 2022a; Skarha et al. 2022; Monteiro et al. 2013; Weinberger et al. 2021; Burkart et al. 2011; Wellenius et al. 2017; Rosenthal et al. 2014; Sung et al. 2013; Metzger et al. 2010; Levy et al. 2015; Desai et al. 2015; Fritze 2020; Rathi et al. 2017, 2021) 01 Certain infectious or parasitic diseases (Saddique et al. 2021; Williams et al. 2020) 02 Neoplasms (Kivimäki et al. 2050; Williams et al. 2020) 05 Endocrine, nutritional or metabolic diseases (Vaidyanathan et al. 2019; Liu et al. 2018b; Weinberger et al. 2021; Williams et al. 2020; Boeke et al. 2010) 06 Mental, behavioural or neurodevelopmental disorders (Williams et al. 2020; Deng et al. 2022; Gao et al. 2020) 08 Diseases of the nervous system (Williams et al. 2020; Hahn et al. 2023) 11 Diseases of the circulatory system (Vaidyanathan et al. 2019; Liu et al. 2018b; Kivimäki et al. 2050; Monteiro et al. 2013; Weinberger et al. 2021; Burkart et al. 2011; Wellenius et al. 2017; Williams et al. 2020; Hahn et al. 2023; Brunetti et al. 2014; Khatana et al. 2022b; Kysely and Huth 2004; Huang et al. 2021; Yin and Wang 2018) 12 Diseases of the respiratory system (Vaidyanathan et al. 2019; Liu et al. 2018b; Monteiro et al. 2013; Williams et al. 2020; Hahn et al. 2023) 16 Diseases of the genitourinary system (Gunasekara et al. 2023; Vaidyanathan et al. 2019; Liu et al. 2018b; Weinberger et al. 2021; Hamilton et al. 2021; Ross et al. 2018; Chu et al. 2022) 18 Pregnancy, childbirth or the puerperium (Tam et al. 2008) 19 Certain conditions originating in the perinatal period (Jiao et al. 2023; Son et al. 2019; Carlson et al. 2021) 20 Developmental anomalies (Williams et al. 2020) 21 Symptoms, signs or clinical findings, not elsewhere classified (Kranc et al. 2021) 22 Injury, poisoning or certain other consequences of external causes (Sen and Nag 2019; Weinberger et al. 2021; Wellenius et al. 2017; Hahn et al. 2023; Bandala et al. 2019; Shire et al. 2020; Saha et al. 2015; Lewandowski et al. 2022; Na et al. 2013; DeMartini et al. 2014; Grundstein et al. 2012; Bethancourt et al. 2021; Bai et al. 2014; Erickson et al. 2019; Hartz et al. 2012; Moore et al. 2017) 23 External causes of morbidity or mortality (Kivimäki et al. 2050; Williams et al. 2020; Cloud et al. 2023) 24 Factors influencing health status or contact with health services (Savitz and Hu 2021) Other (non-categorized in one code) (Spangler et al. 2023a; Kivimäki et al. 2050; Weinberger et al. 2021; Williams et al. 2020; Gao et al. 2020)
HL	Greece	Temperate	22 Injury, poisoning or certain other consequences of external causes (Pantavou et al. 2011; Theoharatos et al. 2010)
HU	Canada, India, Italy, People’s Republic of China, Republic of China (Taiwan), Russia, Sri Lanka, United States	Continental Temperate Tropical	All causes (Lin et al. 2012; Isaksen et al. 2016, 2015; DeVine et al. 2017; Rainham and Smoyer-Tomic 2003; Conti et al. 2007, 2005; Kegel et al. 2021; Calkins et al. 2016; Ho et al. 2017; Arnold et al. 2022) 01 Certain infectious or parasitic diseases (Zhang et al. 2017) 04 Diseases of the immune system (Calkins et al. 2016) 05 Endocrine, nutritional or metabolic diseases (Isaksen et al. 2016, 2015; Calkins et al. 2016; Arnold et al. 2022) 06 Mental, behavioural or neurodevelopmental disorders (Isaksen et al. 2016, 2015; Calkins et al. 2016; Arnold et al. 2022; Zhou et al. 2023) 08 Diseases of the nervous system (Shartova et al. 2018; Isaksen et al. 2016, 2015; Calkins et al. 2016; Arnold et al. 2022) 09 Diseases of the visual system (Zhao et al. 2023) 11 Diseases of the circulatory system (Shartova et al. 2018; Isaksen et al. 2016, 2015; Calkins et al. 2016; Arnold et al. 2022) 12 Diseases of the respiratory system (Isaksen et al. 2016, 2015; Calkins et al. 2016; Arnold et al. 2022; Pan et al. 2019) 16 Diseases of the genitourinary system (Gunasekara et al. 2023; Isaksen et al. 2016, 2015; Arnold et al. 2022) 22 Injury, poisoning or certain other consequences of external causes (Sen and Nag 2019; Isaksen et al. 2016, 2015; Calkins et al. 2016; Bassil et al. 2011) 23 External causes of morbidity or mortality (Isaksen et al. 2016) Other (non-categorized in one code) (Calkins et al. 2016; Mastrangelo et al. 2007; Infusino et al. 2021)
PET	Austria, Bangladesh, Brazil, Croatia, Czech Republic, Germany, Greece, Iran, People's People’s Republic of China, Portugal, Republic of Cyprus, Republic of Korea, Russia, Sweden, Turkey, United States	Arid, Continental, Temperate, Tropical	All causes (Aboubakri et al. 2020; Burkart et al. 2011; Thach et al. 2015; Schroeder et al. 2023; Thorsson et al. 2021; Laschewski and Jendritzky 2002; Pantavou et al. 2021; Muthers et al. 2010a, 2010b; Urban et al. 2019; Matzarakis et al. 2011; Nastos and Matzarakis 2012; Dastoorpoor et al. 2022a; Zaninovic and Matzarakis 2014; Zemtsov et al. 2020; Sharafkhani et al. 2018) 02 Neoplasms (Shiue et al. 2015) 08 Diseases of the nervous system (Shartova et al. 2018; Urban and Kyselý 2014; Dastoorpoor et al. 2022a, 2022b; Ferrari et al. 2015; Lim et al. 2017) 11 Diseases of the circulatory system (Shartova et al. 2018; Urban and Kyselý 2014; Mohammadi and Karimi 2018; Costa et al. 2021; Burkart et al. 2011; Thach et al. 2015; Dastoorpoor et al. 2022a, 2022b; Sharafkhani et al. 2018; Shiue et al. 2016a, 2016b, 2016c; Vasconcelos et al. 2013; Roshan et al. 2022a; Roshan et al. 2022b; Caglak 2022) 12 Diseases of the respiratory system (Thach et al. 2015; Dastoorpoor et al. 2022a; Sharafkhani et al. 2018; Caglak and Matzarakis 2023; Nastos and Matzarakis 2006; Borsi et al. 2021) 11 and 12 Diseases of the circulatory and Diseases of the respiratory system (Muthers et al. 2010a; Silva and Ribeiro 2012) 18 Pregnancy, childbirth or the puerperium (Dastoorpoor et al. 2021) 19 Certain conditions originating in the perinatal period (Dastoorpoor et al. 2021) 21 Symptoms, signs or clinical findings, not elsewhere classified (Thorsson et al. 2021; Nastos and Matzarakis 2008) 22 Injury, poisoning or certain other consequences of external causes (Hartz et al. 2013; Pantavou et al. 2020) 24 Factors influencing health status or contact with health services (Dastoorpoor et al. 2021) Other (non-categorized in one code) (Schroeder et al. 2023)
PMV	Brazil, Greece, Iran	Arid, Temperate	All causes (Aboubakri et al. 2020) 11 Diseases of the circulatory system (Mohammadi and Karimi 2018; Costa et al. 2021; Roshan et al. 2022b) 11 and 12 Diseases of the circulatory and Diseases of the respiratory system (Pantavou et al. 2008) 12 Diseases of the respiratory system (Nastos and Matzarakis 2006)
PT	Austria, Germany, Iran, People’s Republic of China	Arid, Continental, Temperate	11 Diseases of the circulatory system (Roshan et al. 2022b; Kienbacher et al. 2021; Koppe et al. 2011; Schlegel et al. 2020) 11 and 12 Diseases of the circulatory and Diseases of the respiratory system (Leung et al. 2021)
RSI	Argentina	Temperate	All causes (Garin and Bejaran 2003)
SET	Brazil, Greece, Iran	Arid, Temperate	All causes (Aboubakri et al. 2020) 11 Diseases of the circulatory system (Costa et al. 2021) 12 Diseases of the respiratory system (Nastos and Matzarakis 2006)
Tek	Iran	Temperate	11 Diseases of the circulatory system (Mohammadi and Karimi 2018)
TS	Greece	Temperate	22 Injury, poisoning or certain other consequences of external causes (Pantavou et al. 2011)
UTCI	Australia, Bangladesh, Brazil, Czech Republic, Germany, Ghana, Greece, India, Iran, Italy, People’s Republic of China, Poland, Portugal, Republic of Cyprus, Spain, Sweden, The Cambia, United States	Arid, Continental, Temperate, Tropical	All causes (Morabito et al. 2014; Aboubakri et al. 2020; Spangler et al. 2023a; Burkart et al. 2011, 2013, 2016, 2014; Thorsson et al. 2021; Pantavou et al. 2021; Urban et al. 2019; Nastos and Matzarakis 2012; Błażejczyk et al. 2018; Blazejczyk et al. 2022; Chau et al. 2022; Urban et al. 2021; Ghada et al. 2021a, 2021b; Romaszko et al. 2017; Lokys et al. 2018) 01 Certain infectious or parasitic diseases (Burkart et al. 2014) 05 Endocrine, nutritional or metabolic diseases (Romaszko et al. 2022a) 08 Diseases of the nervous system (Urban and Kyselý 2014; Jingesi et al. 2023; Ma et al. 2018) 11 Diseases of the circulatory system (Urban and Kyselý 2014; Santurtun et al. 2020; Burkart et al. 2011, 2014; Jingesi et al. 2023; Skutecki et al. 2019) 11 and 12 Diseases of the circulatory and Diseases of the respiratory system (Kuchcik 2021) 12 Diseases of the respiratory system (Romaszko et al. 2019; Lindner-Cendrowska and Bröde 2021; Romaszko-Wojtowicz et al. 2020; Kruger and Nedel 2023; Fallah Ghalhari et al. 2016) 19 Certain conditions originating in the perinatal period (Nyadanu et al. 2022a; Nyadanu et al. 2023a) 21 Symptoms, signs or clinical findings, not elsewhere classified (Thorsson et al. 2021; Cymes et al. 2021) 22 Injury, poisoning or certain other consequences of external causes (Pantavou et al. 2016; Hartz et al. 2013; Sen and Nag 2019; Krzyzewska et al. 2017) 23 External causes of morbidity or mortality (Ghada et al. 2021b) 24 Factors influencing health status or contact with health services (Nyadanu et al. 2023b, 2022b) Other (non-categorized in one code) (Spangler et al. 2023a; Khodadadi et al. 2022; Bonell et al. 2022)
WBGT	Australia, Czech Republic, India, Qatar, Republic of China (Taiwan), Sri Lanka, Sweden, Tanzania, Thailand, The Cambia, United States	Arid, Continental, Temperate, Tropical	All causes (Lin et al. 2012; Spangler et al. 2023a; Schroeder et al. 2023; Thorsson et al. 2021; Urban et al. 2019) 05 Endocrine, nutritional or metabolic diseases (Sombatsawat et al. 2023) 11 Diseases of the circulatory system (Pradhan et al. 2019; Meshi et al. 2018) 12 Diseases of the respiratory system (Sombatsawat et al. 2023) 16 Diseases of the genitourinary system (Gunasekara et al. 2023) 21 Symptoms, signs or clinical findings, not elsewhere classified (Thorsson et al. 2021; Sombatsawat et al. 2023; Meshi et al. 2018) 22 Injury, poisoning or certain other consequences of external causes (Sen and Nag 2019; Vassil et al. 2020; Lewandowski et al. 2022; Grundstein et al. 2012; Erickson et al. 2019; Sombatsawat et al. 2023; Meshi et al. 2018; Lewandowski and Shaman 2022; Morris et al. 2019; Wallace et al. 2005) Other (non-categorized in one code) (Spangler et al. 2023a; Schroeder et al. 2023; Bonell et al. 2022)
WCT	Brazil, Germany, Ireland, Norway, People’s Republic of China, Republic of Korea, Russia, Sweden, United Kingdom	Continental, Temperate	All causes (Fritze 2022; Carder et al. 2005; Kim et al. 2018) 01 Certain infectious or parasitic diseases (Oh et al. 2021; Kim et al. 2020; Lim et al. 2021) 06 Mental, behavioural or neurodevelopmental disorders (Gao et al. 2020) 08 Diseases of the nervous system (Gill et al. 1988; Kim et al. 2016; Eng and Mercer 2000) 11 Diseases of the circulatory system (Emelina et al. 2015; Carder et al. 2005; Eng and Mercer 2000; Ohlson et al. 1991) 12 Diseases of the respiratory system (Nick et al. 2022) 22 Injury, poisoning or certain other consequences of external causes (Kim et al. 2018) Other (non-categorized in one code) (Gao et al. 2020)

Abbreviations: *ASV* Actual Sensation Vote; *AT* Apparent Temperature; *DI* Discomfort Index; *ET* Effective Temperature; *HI* Heat Index; *HL* Heat Load; *HU* Humidex; *PET* Physiologically Equivalent Temperature; *PMV* Predicted Mean Vote; *PT* Perceived Temperature; *RSI* Relative Strain Index; *SET* Standard Effective Temperature; *Tek* Equivalent Temperature; *TS* Thermal Sensation; *UTCI* Universal Thermal Climate Index; *WBGT* Wet-Bulb Globe Temperature; *WCT* Wind Chill Temperature

Fifty-one countries across six continents- Asia, Africa, North America, South America, Europe, and Oceania- were represented in the associations. About 96% of the associations were reported in studies conducted in North America (n = 448, 39.2%), Europe (n = 399, 34.9%), and Asia (n = 251, 22%) (Online Resource 1–Figure S3). The countries with the highest representation (Online Resource 1–Table S1) were the United States (n = 430, 37.6%), Italy (n = 80, 7%), and the People's Republic of China (n = 69, 6%). Climates considered in the associations were primarily temperate (n = 595, 52.1%) while continental (n = 238, 21%), arid (n = 89, 7.8%), and tropical climates (n = 47, 4.1%) were also examined (Online Resource 1–Figure S4).

**Fig. 2:**
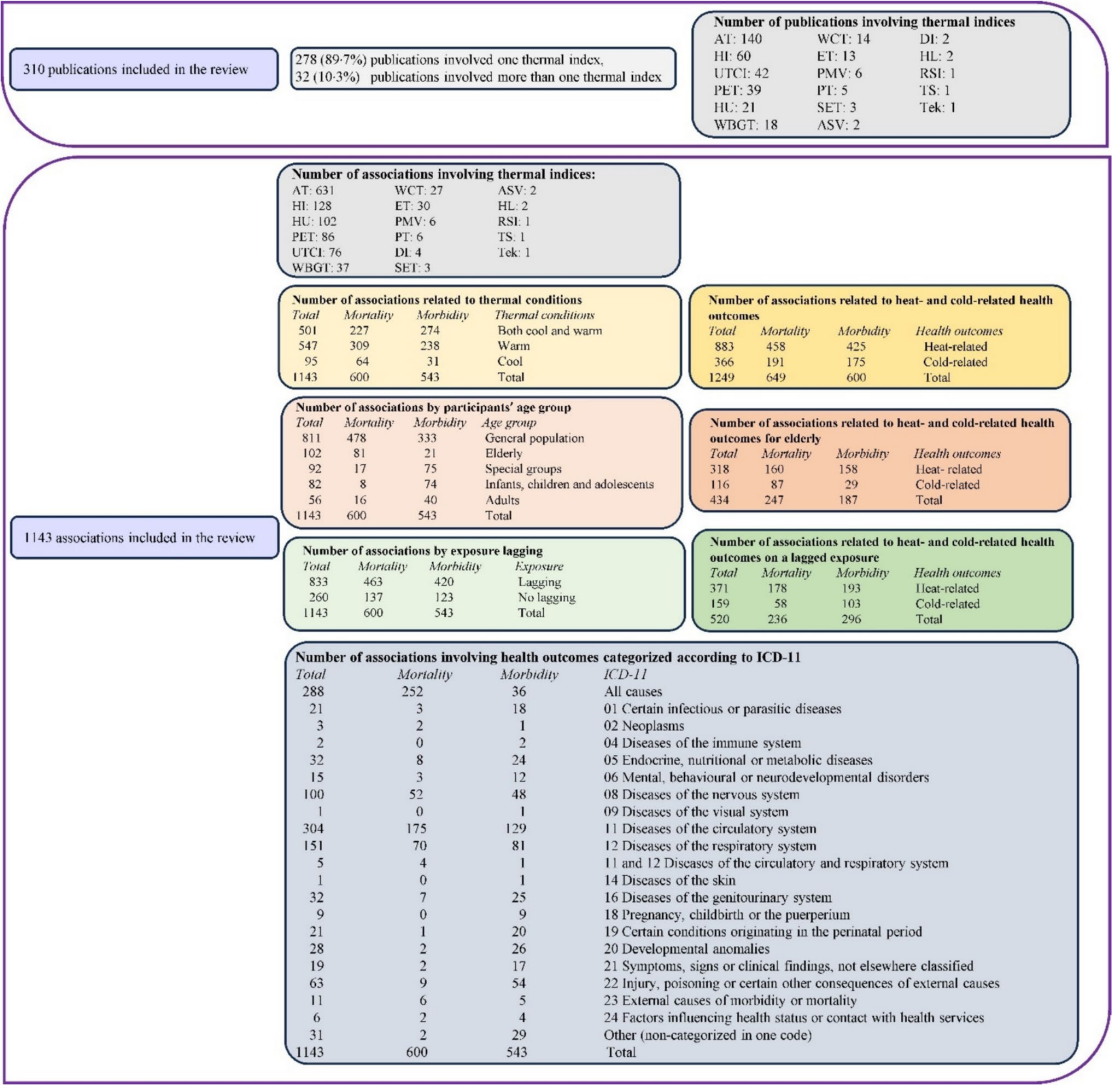
Distribution of publications and associations examining the association between thermal indices and health outcomes. Abbreviations: ASV, Actual Sensation Vote; AT, Apparent Temperature; DI, Discomfort Index; ET, Effective Temperature; HI, Heat Index; HL, Heat Load; HU, Humidex; ICD-11, International Statistical Classification of Diseases and Related Health Problems 11 th Revision; PET, Physiologically Equivalent Temperature; PMV, Predicted Mean Vote; PT, Perceived Temperature; RSI, Relative Strain Index; SET, Standard Effective Temperature; Tek, Equivalent Temperature; TS, Thermal Sensation; UTCI, Universal Thermal Climate Index WBGT, Wet-Bulb Globe Temperature; WCT, Wind Chill Temperature

The associations were examined across a variety of thermal conditions and seasons. Among the 1143 associations analyzed, 43.8% (n = 501) considered both cool and warm thermal conditions, while 47.9% (n = 547) focused exclusively on warm thermal conditions and 8.3% (n = 95) associations examined only cool thermal conditions (Fig. 2). Since some studies assessed both cool and warm thermal conditions for the same health outcome, the total number of associations for heat- and cold-related effects combined (n = 1249) exceeds the overall number of associations (n = 1143). Annual thermal conditions were addressed in 42.3% (n = 484) of the associations. Additionally, 16.4% (n = 188) focused on a single season—winter, spring, summer, or autumn—while 30.7% (n = 351) analyzed summer along with a transitional season, such as spring or autumn. Furthermore, 31% (n = 354) of the associations specifically examined extreme thermal conditions, such as heatwaves or cold spells.

### Thermal indices

A total of seventeen indices were used in the publications included in this systematic review (Fig. 2). Of these, 32 publications (10.3%) used more than one index. The most frequently used index was AT, which appeared in 631 associations (55.2%), followed by HI in 128 (11.2%), PET in 86 (7.5%), and UTCI in 76 associations publications (6.7%) (Online Resource 1–Figure S5**)**.

The indices were estimated using diverse meteorological data sources and type of indices’ measures. In most associations, the indices were estimated using weather stations within the network of official meteorological services or institutions (n = 552, 48.3%). In several associations, the calculation of the indices relied on data derived from airport stations (n = 221, 19.3%) and some on a combination of surface and airport stations (n = 141, 12.3%). In other associations the indices were derived from gridded meteorological data (n = 121, 10.6%), in-situ measurements, data found on websites (i.e., www.weather.org, www.accuweather.com), satellite data, and kriging interpolation (n = 63, 5.5%). The diversity of indices calculation was also evident in the type of indices’ measure. Most associations (n = 607, 53.1%) between indices and health outcomes were estimated using indices’ daily, weekly, and monthly measures of central tendency such as the mean or median value of the indices. Associations based on extreme values, such as maximum or minimum value, were included in 428 (37.5%) associations. Additionally, 50 associations (4.4%) used both measures of central tendency and extreme values.

### Health outcomes

Nearly half of the associations focused on mortality (n = 600, 52.5%), while the other half examined morbidity (n = 543, 47.5%; p-value = 0.0914; Fig. 2). The associations mainly concerned the general population (n = 811, 71%; Fig. 2). Secondarily, mortality was studied specifically among the elderly (n = 81, 13.5% of 600), while morbidity was examined particularly among special population groups (n = 75, 13.9% of 543) including outdoor workers (n = 30, 5.5% of 543), women (n = 23, 4.2% of 543), and athletes (n = 15, 2.8% of 543) as well as among infants, children and adolescents (n = 74, 13.6% of 543). Nevertheless, subgroup analysis for the elderly populations was often provided in the studies.

Mortality data were primarily retrieved from national statistical services (n = 367, 61.2% of 600), health centers including centers for catastrophic injuries or for disease control and prevention (n = 93, 15.5% of 600), and hospitals (n = 75, 12.5% of 600). Morbidity data were obtained from hospitals based on hospital admissions (189, 34.8% of 543) and emergency department visits (n = 115, 21.2% of 543), as well as emergency calls to services such as ambulance (n = 69, 12.7% of 543).

Eighteen categories of specific-cause mortality or morbidity were examined according to the ICD-11 (Fig. 2). The most frequently examined associations were for diseases of the circulatory system (n = 304, 26.6%), followed by all-cause morbidity or mortality (n = 288, 25.2%), diseases of the respiratory system (n = 151, 13.2%), and diseases of the nervous system (n = 100, 8.8%). Among circulatory system diseases, cardiovascular diseases accounted for the majority (n = 164, 53.9% of 304), with coronary heart disease comprising a significant portion (n = 82, 27.6% of 304). For respiratory system diseases, nearly all cases were classified as overall respiratory diseases (n = 150, 99.3% of 151), while cerebrovascular diseases dominated the nervous system category (n = 84, 84% of 100).

The high percentage of associations examining all-cause health outcomes is largely due to those focusing on mortality which primarily concentrated on all-cause health outcomes. The associations focusing on morbidity examined a broader range of ICD-11 categories than those focusing on mortality (18 versus 14). Among mortality associations, 42% (of 600, n = 252) addressed all-cause mortality, with additional focus on diseases of the circulatory system (n = 175, 29.2% of 600) and respiratory system (n = 70, 11.7 of 600%). Conversely, associations focusing on morbidity addressed most frequently diseases of the circulatory (n = 129, 23.8% of 543) and respiratory system (n = 81, 14.9% of 543) (Fig. 2).

### Methods of statistical analysis used in primary studies

The publications included in this systematic review examined the association of thermal indices with health-related outcomes using various statistical methods. Poisson regression or Poisson-family models were used in 508 associations (44.4%). Logistic regression was applied in 238 associations (20.8%), while negative binomial regression was used in 12 associations (1.1%). Linear regression was employed in 50 associations (4.4%). Correlations and tests for comparison of means were conducted in 82 associations (7.2%). Other statistical methods, such as Cox regression models, partial least squares regression, cubic regression, and survival analysis, were used in 222 associations (19.4%). In some cases, the association of thermal indices with health-related outcomes was presented descriptively in Figs. (29 studies, 2.5%).

Effect estimates such as odds ratios, hazard ratios, incidence rate ratios, and relative risk were provided in 53.2% (n = 608) of the associations. Nearly 62% of the associations were adjusted for confounding variables including day of week and holidays, air quality, and participants’ sociodemographic characteristics. Exposure lagging was examined in most of the associations (n = 883, 77.3%), both for mortality (n = 465, 77.2%) and morbidity (n = 420, 77.3%); however, the health outcomes of these associations were reported in 371 of them.

### Outcome of associations

**Fig. 3:**
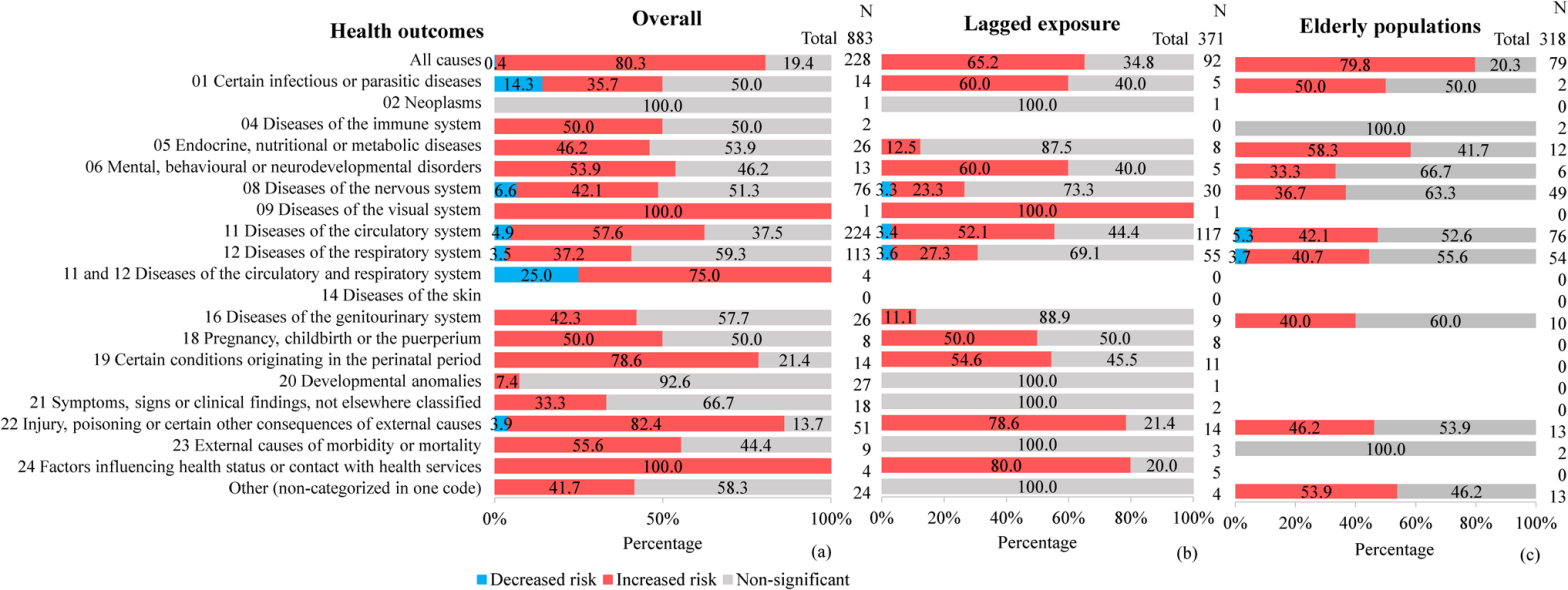
Percentage of associations for heat-related health outcomes, categorized by ICD-11 (11 th revision of the International Classification of Diseases). Results are shown for (**a**) overall population, (**b**) lagged exposure effects, and (**c**) elderly populations

Among the 883 associations focusing on heat-related health outcomes (Fig. 3a), 57.8% (n = 510) indicated an increased risk of adverse health outcomes as thermal indices increased, while 39.3% (n = 347) were non-significant. The increased risk was more pronounced for mortality (71.6% of 458, n = 328) than for morbidity (42.8% of 425, n = 182). This difference was particularly striking for circulatory system diseases, where 77% (n = 107 of 139) of mortality-related analyses showed increased risk, compared to only 28.9% (n = 22 of 85) for morbidity (p-value < 0.001). A similar pattern was observed for respiratory system diseases, where an increased risk was reported in 55.1% (n = 27 of 49) for mortality-focused analysis versus 23.4% (n = 15 of 64) for morbidity (p-value < 0.001). However, the trend was reversed for all-cause morbidity, which had a higher percentage than mortality. In this case, 86.7% (n = 26 of 30) of morbidity-related analyses showed an increased risk, compared to 79.7% (n = 157 of 198) for mortality (p-value < 0.001).

A similar pattern was found for lagged exposure and elderly populations. Among the 371 associations with lagged exposure outcomes (Fig. 3b), 47.7% (n = 177 of 371) suggested an increased risk of adverse health outcomes as indices increased and 50.4% (n = 187 of 371) were non-significant. Mortality showed a higher risk (59%, n = 105 of 178) than morbidity (37.3%, n = 72 of 193; p-value < 0.001), particularly for circulatory system diseases (mortality 70.2%, n = 40 of 57 versus morbidity 35%, n = 21 of 60; p-value = 0.001). Of the 318 associations related to elderly outcomes (Fig. 3c), 50.9% (n = 162 of 318) showed an increased risk of adverse health outcomes as indices increased while 47.2% (n = 150 of 318) were non-significant. Again, mortality exceeded morbidity (71.9%, n = 115 of 160 versus 29.8%, n = 47 of 158; p-value < 0.001), especially for diseases of the circulatory system (mortality 72.2%, n = 26 of 36 versus morbidity 15%, n = 6 of 40; p-value < 0.001) and for diseases of the respiratory system (mortality 63.2%, n = 12 of 19 versus morbidity 28.6%, n = 10 of 35; p-value = 0.0135).

**Fig. 4:**
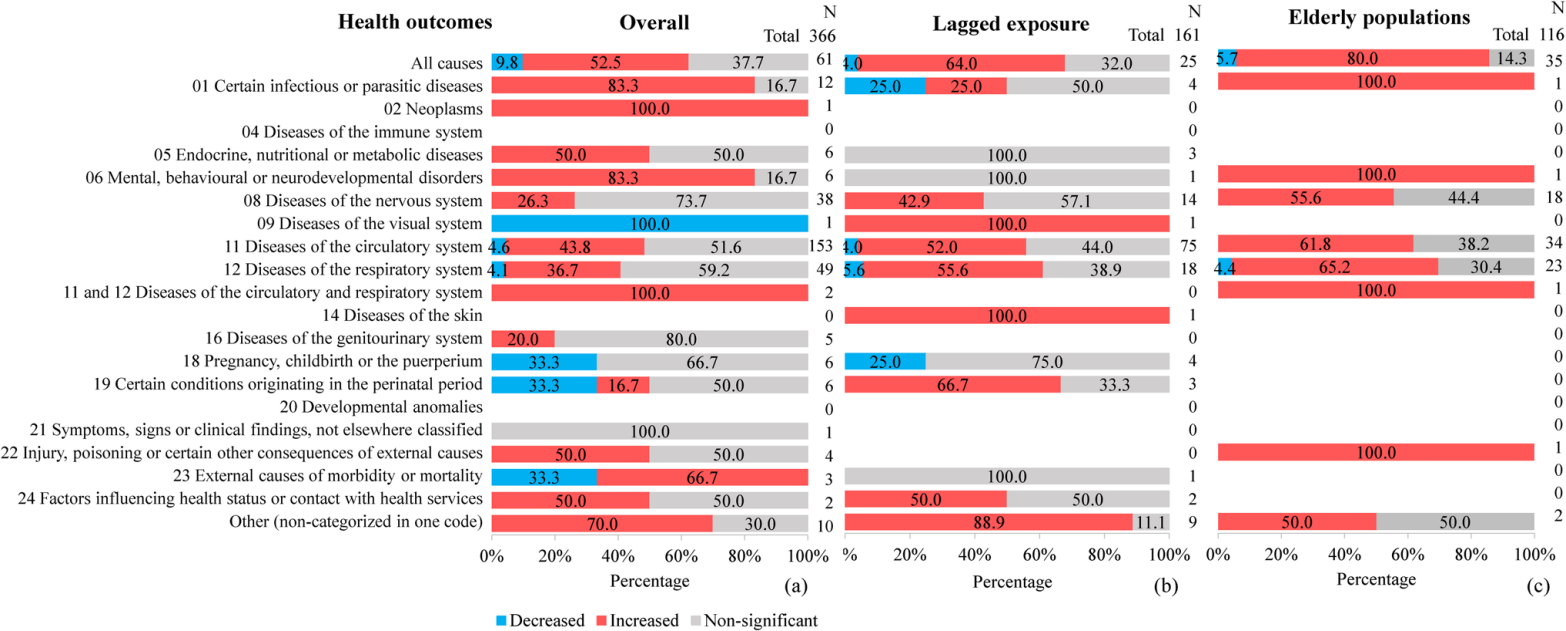
Percentage of associations for cold-related health outcomes, categorized by ICD-11 (11 th revision of the International Classification of Diseases). Results are shown for (**a**) overall population, (**b**) lagged exposure effects, and (**c**) elderly populations

For cold-related health risks (n = 366; Fig. 4a), 44.1% (n = 162 of 366) associations suggested an increased risk of adverse health outcomes as indices decreased, while 49.9% (n = 183 of 366) were non-significant. The risk increase was similar between mortality (46.1%, n = 88 of 191) and morbidity (42.3%, n = 74 of 175). Considering the lagged exposure, among 161 associations (Fig. 4b), 52.8% (n = 85 of 161) indicated an increased risk of adverse health outcomes as indices decreased and 42.1% (n = 69 of 161) were non-significant. Mortality (77.6%, n = 45 of 58) was notably higher than morbidity (38.8%, n = 40 of 103; p-value < 0.001), with circulatory system diseases showing the strongest effect (mortality 87.5%, n = 28 of 32; morbidity 25.6%, n = 11; p-value = 0.001). Of the 116 elderly-related associations (Fig. 4c), 68.1% (n = 79 of 116) suggested increased risk as indices decreased, with 29.3% (n = 34 of 116) being non-significant. Mortality and morbidity percentages of associations suggesting an increased risk were statistically similar (mortality 72.4%, n = 63 of 87; morbidity 55.2%, n = 16 of 29; p-value = 0.0852).

### Quality assessment

The methodological quality of the 310 publications included in our systematic review, as assessed using the NIH Quality Assessment Tool, varied widely (Online Resource 2 − Table S2). The median score was 12, with an interquartile range of 11 to 13. Common limitations included the lack of sample size justification, power analysis, or of reporting variance and effect estimates (n = 241, 77.7%), as well as the failure to measure and statistically adjust for key potential confounding variables that could influence the relationship between exposure and outcome (n = 123, 39.7%).

## Discussion

This systematic review, the first of its kind, summarizes the evidence on the relationship between thermal indices and health outcomes. It provides a structured overview of how different thermal indices have been used in health research, identifies gaps in the literature, and highlights methodological challenges. The findings demonstrate a growing scientific interest over time in using thermal indices to quantify the effects of thermal environments on human health. However, the distribution of research across continents and climate zones is uneven. Studies from the United States and temperate climates dominate the literature, while there is relatively less research in tropical regions, despite the high population density in both tropical and temperate zones (Klinger and Ryan 2022).

Most of the studies included in this systematic review focused on heat-related health risks, likely driven by concerns over climate change and rising global temperatures. However, there is some evidence that cold temperatures are linked to higher mortality rates compared to heat. For instance, Gasparrini et al. (Gasparrini et al. 2015) and Chigozie et al. (Chigozie et al. 2022) found that cold-related mortality, especially among older adults and those with cardiovascular conditions, surpasses the mortality associated with heat. The multi-country study by Gasparrini et al. (Gasparrini et al. 2015) further highlighted that most temperature-related deaths occur during moderately cold days, with a particularly strong trend observed in Mediterranean countries.

In terms of the indices used, this systematic review found that the AT is the most frequently employed index, in contrast to earlier reviews that documented the widespread use of the thermo-physiological indices PET, Predicted Mean Vote, UTCI, and Standard Effective Temperature for assessing outdoor thermal perception (Potchter et al. 2018). This discrepancy could be explained by the high number of studies conducted in the United States (where AT and HI are commonly used by the National Weather Service), as well as the simplicity of their calculation. Nonetheless, despite being more recent, the thermo-physiological indices PET and UTCI were also frequently employed, following AT and HI in terms of frequency of use. PET and UTCI are considered more suitable for human biometeorological evaluations as they account for the energy exchange between the environment and the human body (Matzarakis 2021). Notably, UTCI has been recently incorporated into forecasting procedures at several weather institutes and at the European Centre for Medium-Range Forecasts (Napoli et al. 2021).

This systematic review also found that both mortality and morbidity were equally represented in the associations examined. The most examined outcomes were all-cause morbidity or mortality and all-cause circulatory and respiratory diseases, followed by cerebrovascular diseases and coronary heart diseases. However, other health conditions were underrepresented. Additionally, there was a notable lack of research on thermal indices and vector-borne diseases, which are expected to spread more due to climate change. Most studies indicated an increased risk of heat-related health outcomes as indices rose in warm thermal conditions or fell in cold ones, although the patterns for mortality and morbidity varied. Mortality risk was significantly higher for circulatory and respiratory diseases while morbidity showed a higher percentage for all-cause outcomes.

There was significant heterogeneity in methods to analyze the association between thermal indices and health outcomes. Different metric measures, such as mean, maximum, or minimum values of the indices, raise concerns about potential inconsistencies in calculating effect sizes. While some suggest that these variations exhibit similar predictive abilities, allowing for the combination of studies with different metrics (Bhaskaran et al. 2009), other studies indicate that maximum and mean values are associated with higher relative risks of morbidity and mortality compared to minimum values of the same indices (Spangler et al. 2023b). Furthermore, extreme thermal conditions seem to have a greater impact on health outcomes compared to non-extreme conditions (Heo and Bell 2019). While daily data are typically used, some studies have suggested that weekly data can reasonably estimate short-term exposure–response relationships, while bi-weekly or monthly data are more suited for long-term exposure assessment (Ebi 2024; Ballester et al. 2024).

Many studies employed inadequate or overly simplistic statistical approaches, often failing to adjust for confounders. Additionally, frequently statistically significant results and effect sizes were reported, while non-significant findings were often omitted. In some cases, the lag period of the effect was extended until statistical significance was achieved. This selective reporting likely introduces bias and compromises the validity of any meta-analysis conducted on the available data.

This systematic review represents the most comprehensive review to date on the associations of thermal indices with population health. It provides insights that support the use of thermal indices as a more integrated approach to accurately estimating the thermal environment for heat-related prevention recommendations and guidance, both at an individual level and for public health initiatives. However, this study has certain limitations. The search query may not have included all possible search terms for existing thermal indices in the literature. The selected terms focused on indices commonly used in biometeorological studies and weather services. Nonetheless, given the broad scope of our search-incorporating general thermal and health-related terms, it likely captured studies relevant to these indices despite their omission as specific search terms. Significant differences in terms of thermal indices, health outcomes, study designs, or inconsistent and incomplete reporting made it inappropriate to quantitatively synthesize the data in a meta-analysis. This highlights the need for standardization in methodology and reporting. Key recommendations that would enhance comparisons of results across different regions, climates, and studies include:

- Use of standardized thermal indices: thermal indices such as UTCI and PET apply across all climates and thermal conditions (i.e., warm, cool).
- Preference for thermo-physiological indices: thermo-physiological indices are better suited for human biometeorological evaluations, as they account for the complex interactions between the human body and the environment (Matzarakis 2024). These indices should be prioritized for studies examining the effects of thermal environments on human health.
- Robust statistical methods: it is essential to use advanced and robust statistical methods that account for confounding factors and provide reliable effect sizes. These methods include multi-variable regression models (e.g., Poisson, negative binomial), as well as time-series analysis techniques.
- Systematic and detailed reporting of sample size and characteristics: reporting sample sizes and participant characteristics (e.g., age, gender, health status) allows for more precise comparisons across studies.
- Standardized reporting of health outcomes: health outcomes should be systematically categorized and reported using internationally recognized classifications (e.g., ICD codes); furthermore, the methods used for measuring health outcomes (e.g., hospital admissions, mortality records) should be reported.
- Comprehensive presentation of results: effect sizes should be systematically reported for all associations, including non-significant results, and standardized metrics, such as mean, median, maximum and minimum values of thermal indices, should be consistently used for comparisons. This approach can enhance the accuracy of findings and reduce the risk of selective reporting bias.

## Conclusions

Thermal indices, which consider multiple meteorological factors, offer a valuable tool for public health planning and are especially relevant in the context of climate change. These indices are well-established methods for assessing thermal environment and its effects on human health, and can serve as effective operational tools for developing public health protection policies. Summarizing the existing evidence on thermal indices is expected to raise awareness among public health professionals and policymakers, promoting their wider application in public health initiatives.

This systematic review reveals significant associations between thermal indices and health outcomes, highlighting the broad and multidimensional impact of thermal environment on human health. However, the diversity in methodologies and reporting across the literature complicates direct comparisons and limits the ability to synthesize data effectively. This problem emphasizes the need for standardization in future research, particularly in how thermal indices, health outcomes, and their associations are measured and reported. Moreover, expanding the range of health outcomes studied, especially across different climates, is essential in order to fully understand the relationship between thermal stress and health. This broader approach will help establish associations and provide a more comprehensive picture of how climate impacts health across various geographic regions.

As climate change continues, integrated approaches that monitor the relationship between thermal conditions and health outcomes, including the use of thermal indices, are essential for developing, implementing, and evaluating effective health policies.

## Supplementary Information

Below is the link to the electronic supplementary material.

Supplementary file1 (DOCX 609 KB)

Supplementary file2 (XLSX 213 KB)

## Data Availability

Most of the data and the list of all meta-analyses not selected for data extraction are provided in the supplemental material.
