# Supplementary material for: Thermal indices for assessing the impact of outdoor thermal environments on human health: a systematic review of epidemiological studies
Source: Int J Biometeorol. 2025 Jun 2;69(8):1843–66. doi: 10.1007/s00484-025-02948-x (PMC12287235; doi:10.1007/s00484-025-02948-x)
Supplement: Supplementary file 1 — Supplementary file1 (DOCX 609 KB) [file 484_2025_2948_MOESM1_ESM.docx]

**Supplemental Material**

**International Journal of Biometeorology**

**Thermal indices for assessing the impact of outdoor thermal environments on human health: a systematic review of epidemiological studies**

Katerina Pantavou^a,b^, Adrien Fillon^c^, Lunzheng Li^c^, Zacharias Maniadis^c,d^, and Georgios K. Nikolopoulos^a^

^a^Medical School, University of Cyprus, 1678, Nicosia, Cyprus

^b^Institute for Environmental Research and Sustainable Development, National Observatory of Athens, 15236 Athens, Greece

^c^Department of Economics, University of Cyprus, 1678, Nicosia, Cyprus

^d^Economics Department, University of Southampton, SO17 1BJ, Southampton, United Kingdom

Correspondence to: Katerina Pantavou, [kpantav@noa.gr](mailto:kpantav@noa.gr)

**Contents**

[**Search strategy** 3](#_Toc194270905)

[**PRISMA Checklist** 4](#_Toc194270906)

[**Studies and associations** 6](#_Toc194270907)

[**Figure S1** Distribution of publications (n=310) examining the association between thermal indices and health outcomes over time 6](#_Toc194270908)

[**Figure S2** Distribution of publications (n=310) examining the association between thermal indices and health outcomes across different scientific journal disciplines 6](#_Toc194270909)

[**Figure S3** Distribution of associations (n=1143) examining the effect of thermal indices on health outcomes across different continents 7](#_Toc194270910)

[**Table S1** Distribution of associations (n=1143) examining the effects of thermal indices on health outcomes across different countries 8](#_Toc194270911)

[**Figure S4** Distribution of associations (n=1143) examining the effect of thermal indices on health outcomes across different climates. Climate classifications include Temperate (Cfa, Cfb, Cfc, Csa, Csb, Csc, Cwa, Cwb), Continental (Dfa, Dfb, Dfc, Dfd), Arid (BSh, BSk, BWh, BWk), and Tropical (Af, Am, As, Aw). 9](#_Toc194270912)

[**Figure S5** Distribution of thermal indices used in 1143 associations examining the effect of thermal indices on health outcomes. The figure presents the percentage (%) of associations for each thermal index. 10](#_Toc194270913)

# **Search strategy**

**Pubmed**

(heat[Title/Abstract] OR cold[Title/Abstract] OR cool[Title/Abstract] OR warm[Title/Abstract] OR "thermal environment"[Title/Abstract] OR "thermal condition"[Title/Abstract] OR weather[Title/Abstract] OR climate[Title/Abstract]) AND ("thermal index"[Title/Abstract] OR "thermal indices"[Title/Abstract] OR "biometeorological index"[Title/Abstract] OR "biometeorological indices"[Title/Abstract] OR "equivalent temperature"[Title/Abstract] OR PET[Title/Abstract] OR "universal thermal climate index"[Title/Abstract] OR UTCI[Title/Abstract] OR PMV[Title/Abstract] OR "predicted mean vote"[Title/Abstract] OR "wind chill"[Title/Abstract] OR "heat index"[Title/Abstract] OR "apparent temperature"[Title/Abstract] OR humidex[Title/Abstract] OR "effective temperature" OR "perceived temperature"[Title/Abstract]) AND (mortality[Title/Abstract] OR death[Title/Abstract] OR morbidity[Title/Abstract] OR hospital[Title/Abstract] OR emergency[Title/Abstract] OR health[Title/Abstract] OR exposure[Title/Abstract] OR exhaustion[Title/Abstract] OR illness[Title/Abstract] OR disease[Title/Abstract]) Filters: Humans

**Scopus**

TITLE-ABS-KEY ( heat OR cold OR cool OR warm OR "thermal environment" OR "thermal condition" OR weather OR climate ) AND TITLE-ABS-KEY ( "thermal index" OR "thermal indices" OR "biometeorological index" OR "biometeorological indices" OR "equivalent temperature" OR pet OR "universal thermal climate index" OR utci OR pmv OR "predicted mean vote" OR "wind chill" OR "heat index" OR "apparent temperature" OR humidex OR "effective temperature" OR "perceived temperature" ) AND TITLE-ABS-KEY ( mortality OR death OR morbidity OR hospital OR emergency OR health OR exposure OR exhaustion OR illness OR disease ) AND ( LIMIT-TO ( EXACTKEYWORD , "Human" ) OR LIMIT-TO ( EXACTKEYWORD , "Humans" ) )

**Web of Science**

(AB=(heat) OR AB=(cold) OR AB=(cool) OR AB=(warm) OR AB=(thermal environment) OR AB=(thermal condition) OR AB=(weather) OR AB=(climate)) AND (AB=(thermal index) OR AB=(thermal indices) OR AB=(biometeorological index) OR AB=(biometeorological indices) OR AB=(equivalent temperature) OR AB=(PET) OR AB=(universal thermal climate index) OR AB=(UTCI) OR AB=(PMV) OR AB=(predicted mean vote) OR AB=("wind chill") OR AB=("heat index") OR AB=("apparent temperature") OR AB=(humidex) OR AB=("effective temperature") OR AB=(“perceived temperature”)) AND (AB=(mortality) OR AB=( death) OR AB=(morbidity) OR AB=(hospital) OR AB=( emergency) OR AB=( health) OR AB=( exposure) OR AB=( exhaustion) OR AB=( illness) OR AB=( disease)) Refined by: and Entomology or Zoology or Plant Sciences or Fisheries (Exclude – Research Areas)

# **PRISMA Checklist**

| **Section and Topic** | **Item #** | **Checklist item** | **Location where item is reported** |
| --- | --- | --- | --- |
| **TITLE** | | |  |
| Title | 1 | Identify the report as a systematic review. | Title |
| **ABSTRACT** | | |  |
| Abstract | 2 | See the PRISMA 2020 for Abstracts checklist. | Abstract |
| **INTRODUCTION** | | |  |
| Rationale | 3 | Describe the rationale for the review in the context of existing knowledge. | Paragraphs 1 to 4 |
| Objectives | 4 | Provide an explicit statement of the objective(s) or question(s) the review addresses. | Last paragraph |
| **METHODS** | | |  |
| Eligibility criteria | 5 | Specify the inclusion and exclusion criteria for the review and how studies were grouped for the syntheses. | Methods/ Search strategy and selection criteria |
| Information sources | 6 | Specify all databases, registers, websites, organisations, reference lists and other sources searched or consulted to identify studies. Specify the date when each source was last searched or consulted. | Methods/ Search strategy and selection criteria |
| Search strategy | 7 | Present the full search strategies for all databases, registers and websites, including any filters and limits used. | Additional file 1 – Search strategy |
| Selection process | 8 | Specify the methods used to decide whether a study met the inclusion criteria of the review, including how many reviewers screened each record and each report retrieved, whether they worked independently, and if applicable, details of automation tools used in the process. | Methods/ Search strategy and selection criteria |
| Data collection process | 9 | Specify the methods used to collect data from reports, including how many reviewers collected data from each report, whether they worked independently, any processes for obtaining or confirming data from study investigators, and if applicable, details of automation tools used in the process. | Methods/ Search strategy and selection criteria |
| Data items | 10a | List and define all outcomes for which data were sought. Specify whether all results that were compatible with each outcome domain in each study were sought (e.g. for all measures, time points, analyses), and if not, the methods used to decide which results to collect. | Methods/ Data extraction and analysis |
|  | 10b | List and define all other variables for which data were sought (e.g. participant and intervention characteristics, funding sources). Describe any assumptions made about any missing or unclear information. | Methods and Funding |
| Study risk of bias assessment | 11 | Specify the methods used to assess risk of bias in the included studies, including details of the tool(s) used, how many reviewers assessed each study and whether they worked independently, and if applicable, details of automation tools used in the process. | Methods/ Data extraction and analysis |
| Effect measures | 12 | Specify for each outcome the effect measure(s) (e.g. risk ratio, mean difference) used in the synthesis or presentation of results. | Non-applicable |
| Synthesis methods | 13a | Describe the processes used to decide which studies were eligible for each synthesis (e.g. tabulating the study intervention characteristics and comparing against the planned groups for each synthesis (item #5)). | Non-applicable |
|  | 13b | Describe any methods required to prepare the data for presentation or synthesis, such as handling of missing summary statistics, or data conversions. | Non-applicable |
|  | 13c | Describe any methods used to tabulate or visually display results of individual studies and syntheses. | Methods/ Data extraction and analysis |
|  | 13d | Describe any methods used to synthesize results and provide a rationale for the choice(s). If meta-analysis was performed, describe the model(s), method(s) to identify the presence and extent of statistical heterogeneity, and software package(s) used. | Methods/ Data extraction and analysis |
|  | 13e | Describe any methods used to explore possible causes of heterogeneity among study results (e.g. subgroup analysis, meta-regression). | Non-applicable |
|  | 13f | Describe any sensitivity analyses conducted to assess robustness of the synthesized results. | Non-applicable |
| Reporting bias assessment | 14 | Describe any methods used to assess risk of bias due to missing results in a synthesis (arising from reporting biases). | Methods/ Data extraction and analysis |
| Certainty assessment | 15 | Describe any methods used to assess certainty (or confidence) in the body of evidence for an outcome. | Non-applicable |
| **RESULTS** | | |  |
| Study selection | 16a | Describe the results of the search and selection process, from the number of records identified in the search to the number of studies included in the review, ideally using a flow diagram. | Results/ Studies and associations |
|  | 16b | Cite studies that might appear to meet the inclusion criteria, but which were excluded, and explain why they were excluded. | Results/ Studies and associations |
| Study characteristics | 17 | Cite each included study and present its characteristics. | Table 1 |
| Risk of bias in studies | 18 | Present assessments of risk of bias for each included study. | Table S2 |
| Results of individual studies | 19 | For all outcomes, present, for each study: (a) summary statistics for each group (where appropriate) and (b) an effect estimate and its precision (e.g. confidence/credible interval), ideally using structured tables or plots. | Figures 2 to 4 |
| Results of syntheses | 20a | For each synthesis, briefly summarise the characteristics and risk of bias among contributing studies. | Figures 3 and 4, and Results/ Quality assessment |
|  | 20b | Present results of all statistical syntheses conducted. If meta-analysis was done, present for each the summary estimate and its precision (e.g. confidence/credible interval) and measures of statistical heterogeneity. If comparing groups, describe the direction of the effect. | Results/ Outcome of associations |
|  | 20c | Present results of all investigations of possible causes of heterogeneity among study results. | Figures 3 and 4 |
|  | 20d | Present results of all sensitivity analyses conducted to assess the robustness of the synthesized results. | Figures 3 and 4 |
| Reporting biases | 21 | Present assessments of risk of bias due to missing results (arising from reporting biases) for each synthesis assessed. | Non-applicable |
| Certainty of evidence | 22 | Present assessments of certainty (or confidence) in the body of evidence for each outcome assessed. | Non-applicable |
| **DISCUSSION** | | |  |
| Discussion | 23a | Provide a general interpretation of the results in the context of other evidence. | Paragraphs 2 and 3 |
|  | 23b | Discuss any limitations of the evidence included in the review. | Paragraphs 5 and 6 |
|  | 23c | Discuss any limitations of the review processes used. | Paragraph 7 |
|  | 23d | Discuss implications of the results for practice, policy, and future research. | Paragraph 7 |
| **OTHER INFORMATION** | | |  |
| Registration and protocol | 24a | Provide registration information for the review, including register name and registration number, or state that the review was not registered. | Methods and Funding |
|  | 24b | Indicate where the review protocol can be accessed, or state that a protocol was not prepared. | Methods and Funding |
|  | 24c | Describe and explain any amendments to information provided at registration or in the protocol. | Non-applicable |
| Support | 25 | Describe sources of financial or non-financial support for the review, and the role of the funders or sponsors in the review. | Funding |
| Competing interests | 26 | Declare any competing interests of review authors. | Declaration of Interests |
| Availability of data, code and other materials | 27 | Report which of the following are publicly available and where they can be found: template data collection forms; data extracted from included studies; data used for all analyses; analytic code; any other materials used in the review. | Data sharing |

*From:*  Page MJ, McKenzie JE, Bossuyt PM, Boutron I, Hoffmann TC, Mulrow CD, et al. The PRISMA 2020 statement: an updated guideline for reporting systematic reviews. BMJ 2021;372:n71. doi: 10.1136/bmj.n71

For more information, visit: <http://www.prisma-statement.org/>

**Studies and associations**

## **Figure S1** Distribution of publications (n=310) examining the association between thermal indices and health outcomes over time

## **Figure S2** Distribution of publications (n=310) examining the association between thermal indices and health outcomes across different scientific journal disciplines

## **Figure S3** Distribution of associations (n=1143) examining the effect of thermal indices on health outcomes across different continents

## **Table S1** Distribution of associations (n=1143) examining the effects of thermal indices on health outcomes across different countries

| **Country** | **Freq.** | **Percent** | **Country** | **Freq.** | **Percent** |
| --- | --- | --- | --- | --- | --- |
| USA | 430 | 37.6 | Kazakhstan | 8 | 0.7 |
| Italy | 80 | 7.0 | Switzerland | 8 | 0.7 |
| People's Republic of China | 69 | 6.0 | Slovenia | 7 | 0.6 |
| Spain | 56 | 4.9 | Australia | 6 | 0.5 |
| Iran | 45 | 3.9 | Austria | 6 | 0.5 |
| Czech Republic | 44 | 3.9 | Japan | 6 | 0.5 |
| Republic of Korea | 38 | 3.3 | South Africa | 4 | 0.4 |
| Republic of China (Taiwan) | 34 | 3.0 | Sri Lanka | 4 | 0.4 |
| Greece | 25 | 2.2 | Vietnam | 4 | 0.4 |
| Brazil | 23 | 2.0 | Mexico | 3 | 0.3 |
| Sweden | 21 | 1.8 | Norway | 3 | 0.3 |
| Germany | 20 | 1.8 | Republic of Cyprus | 3 | 0.3 |
| Russia | 18 | 1.6 | Tanzania | 3 | 0.3 |
| Canada | 15 | 1.3 | Turkey | 3 | 0.3 |
| Thailand | 15 | 1.3 | Argentina | 2 | 0.2 |
| Denmark | 14 | 1.2 | Ghana | 2 | 0.2 |
| Portugal | 14 | 1.2 | Israel | 2 | 0.2 |
| United Kingdom | 14 | 1.2 | Malaysia | 2 | 0.2 |
| Multicountry | 13 | 1.1 | The Cambia | 2 | 0.2 |
| Hungary | 11 | 1.0 | Chile | 1 | 0.1 |
| India | 11 | 1.0 | Croatia | 1 | 0.1 |
| Poland | 11 | 1.0 | Pakistan | 1 | 0.1 |
| France | 10 | 0.9 | Qatar | 1 | 0.1 |
| Ireland | 10 | 0.9 | Serbia | 1 | 0.1 |
| Bangladesh | 9 | 0.8 | Tunis | 1 | 0.1 |
| Finland | 9 | 0.8 |  |  |  |

## **Figure S4** Distribution of associations (n=1143) examining the effect of thermal indices on health outcomes across different climates. Climate classifications include Temperate (Cfa, Cfb, Cfc, Csa, Csb, Csc, Cwa, Cwb), Continental (Dfa, Dfb, Dfc, Dfd), Arid (BSh, BSk, BWh, BWk), and Tropical (Af, Am, As, Aw)

## **Figure S5** Distribution of thermal indices used in 1143 associations examining the effect of thermal indices on health outcomes. The figure presents the percentage (%) of associations for each thermal index
